# Differential privacy preserved federated transfer learning for multi-institutional ^68^Ga-PET image artefact detection and disentanglement

**DOI:** 10.1007/s00259-023-06418-7

**Published:** 2023-09-08

**Authors:** Isaac Shiri, Yazdan Salimi, Mehdi Maghsudi, Elnaz Jenabi, Sara Harsini, Behrooz Razeghi, Shayan Mostafaei, Ghasem Hajianfar, Amirhossein Sanaat, Esmail Jafari, Rezvan Samimi, Maziar Khateri, Peyman Sheikhzadeh, Parham Geramifar, Habibollah Dadgar, Ahmad Bitrafan Rajabi, Majid Assadi, François Bénard, Alireza Vafaei Sadr, Slava Voloshynovskiy, Ismini Mainta, Carlos Uribe, Arman Rahmim, Habib Zaidi

**Affiliations:** 1 Division of Nuclear Medicine and Molecular Imaging, Geneva University Hospital, CH-1211 Geneva, Switzerland; 2 Department of Cardiology, Inselspital, University of Bern, Bern, Switzerland; 3 Rajaie Cardiovascular Medical and Research Center, Iran University of Medical Sciences, Tehran, Iran; 4 Research Center for Nuclear Medicine, Shariati Hospital, Tehran University of Medical Sciences, Tehran, Iran; 5 BC Cancer Research Institute, Vancouver, BC Canada; 6 Department of Computer Science, University of Geneva, Geneva, Switzerland; 7 Division of Clinical Geriatrics, Department of Neurobiology, Care Sciences and Society, Karolinska Institutet, Stockholm, Sweden; 8 Department of Medical Epidemiology and Biostatistics, Karolinska Institute, Stockholm, Sweden; 9 The Persian Gulf Nuclear Medicine Research Center, Department of Nuclear Medicine, Molecular Imaging, and Theranostics, Bushehr Medical University Hospital, School of Medicine, Bushehr University of Medical Sciences, Bushehr, Iran; 10 Department of Medical Radiation Engineering, Shahid Beheshti University, Tehran, Iran; 11 Department of Medical Radiation Engineering, Science and Research Branch, Islamic Azad University, Tehran, Iran; 12 Department of Nuclear Medicine, Imam Khomeini Hospital Complex, Tehran University of Medical Sciences, Tehran, Iran; 13 Cancer Research Center, Razavi Hospital, Imam Reza International University, Mashhad, Iran; 14 Echocardiography Research Center, Rajaie Cardiovascular Medical and Research Center, Iran University of Medical Sciences, Tehran, Iran; 15 Department of Radiology, University of British Columbia, Vancouver, BC Canada; 16 Institute of Pathology, RWTH Aachen University Hospital, Aachen, Germany; 17 Department of Public Health Sciences, College of Medicine, The Pennsylvania State University, Hershey, PA 17033 USA; 18 Molecular Imaging and Therapy, BC Cancer, Vancouver, BC Canada; 19 Department of Integrative Oncology, BC Cancer Research Institute, Vancouver, BC Canada; 20 Department of Physics and Astronomy, University of British Columbia, Vancouver, Canada; 21 Geneva University Neuro Center, Geneva University, Geneva, Switzerland; 22 Department of Nuclear Medicine and Molecular Imaging, University of Groningen, University Medical Center Groningen, Groningen, Netherlands; 23 Department of Nuclear Medicine, University of Southern Denmark, Odense, Denmark

**Keywords:** PET/CT, Artefacts, Privacy, Deep learning, Federated learning

## Abstract

**Purpose:**

Image artefacts continue to pose challenges in clinical molecular imaging, resulting in misdiagnoses, additional radiation doses to patients and financial costs. Mismatch and halo artefacts occur frequently in gallium-68 (^68^Ga)-labelled compounds whole-body PET/CT imaging. Correcting for these artefacts is not straightforward and requires algorithmic developments, given that conventional techniques have failed to address them adequately. In the current study, we employed differential privacy-preserving federated transfer learning (FTL) to manage clinical data sharing and tackle privacy issues for building centre-specific models that detect and correct artefacts present in PET images.

**Methods:**

Altogether, 1413 patients with ^68^Ga prostate-specific membrane antigen (PSMA)/DOTA-TATE (TOC) PET/CT scans from 3 countries, including 8 different centres, were enrolled in this study. CT-based attenuation and scatter correction (CT-ASC) was used in all centres for quantitative PET reconstruction. Prior to model training, an experienced nuclear medicine physician reviewed all images to ensure the use of high-quality, artefact-free PET images (421 patients’ images). A deep neural network (modified U2Net) was trained on 80% of the artefact-free PET images to utilize centre-based (CeBa), centralized (CeZe) and the proposed differential privacy FTL frameworks. Quantitative analysis was performed in 20% of the clean data (with no artefacts) in each centre. A panel of two nuclear medicine physicians conducted qualitative assessment of image quality, diagnostic confidence and image artefacts in 128 patients with artefacts (256 images for CT-ASC and FTL-ASC).

**Results:**

The three approaches investigated in this study for ^68^Ga-PET imaging (CeBa, CeZe and FTL) resulted in a mean absolute error (MAE) of 0.42 ± 0.21 (*CI* 95%: 0.38 to 0.47), 0.32 ± 0.23 (*CI* 95%: 0.27 to 0.37) and 0.28 ± 0.15 (*CI* 95%: 0.25 to 0.31), respectively. Statistical analysis using the Wilcoxon test revealed significant differences between the three approaches, with FTL outperforming CeBa and CeZe (*p*-value < 0.05) in the clean test set. The qualitative assessment demonstrated that FTL-ASC significantly improved image quality and diagnostic confidence and decreased image artefacts, compared to CT-ASC in ^68^Ga-PET imaging. In addition, mismatch and halo artefacts were successfully detected and disentangled in the chest, abdomen and pelvic regions in ^68^Ga-PET imaging.

**Conclusion:**

The proposed approach benefits from using large datasets from multiple centres while preserving patient privacy. Qualitative assessment by nuclear medicine physicians showed that the proposed model correctly addressed two main challenging artefacts in ^68^Ga-PET imaging. This technique could be integrated in the clinic for ^68^Ga-PET imaging artefact detection and disentanglement using multicentric heterogeneous datasets.

**Supplementary Information:**

The online version contains supplementary material available at 10.1007/s00259-023-06418-7.

## Introduction

Artefacts in medical imaging are structures that appear in the image but are not present in the patient’s body [1–4]. In whole-body positron emission tomography (PET) imaging, various artefacts can occur for numerous reasons, including the PET image itself and the propagation of artefacts from complementary imaging modalities, such as computed tomography (CT) or magnetic resonance imaging (MRI) in PET/CT and PET/MRI scanners, respectively [1–6]. Attenuation and scatter correction (ASC) during PET image reconstruction are the main steps in which these artefacts can occur [6–8]. In addition, ASC is applied to compensate for photon attenuation and Compton scattering, which is required to achieve quantitative PET imaging [9, 10]. Halo and mismatch artefacts are the most common in PET imaging of gallium-68 (^68^Ga)-labelled radiopharmaceuticals [1–3, 11, 12]. These artefacts can be easily missed if they are not prominent, but if they are highly effective, they can cause a corrupted image that often requires repeated scans [6, 9, 10]. Nevertheless, in most cases, the repeated scan will not rectify these artefacts, as they are unavoidable in certain cases [1–4, 6, 9, 10].

Mismatch artefacts can occur when there is a discrepancy between PET and anatomical imaging (CT or MRI) and can be caused by voluntary and involuntary organ movement [6, 10, 13–15]. An additional device for mismatch tracking or deformable image registration could partially correct this artefact [13, 14]. Mismatch artefacts result in missing, mislocalization and incorrect quantification of malignant lesions in PET images [6, 10]. This might cause misdiagnosis and change patient management [13, 14, 16]. Different CT acquisition protocols have been proposed to mitigate lung-diaphragm interface mismatch, including end-expiration, mid-expiration and respiratory averaged CT using cinematic CT (4D CT). End-expiration CT acquisition has been suggested to enable better assessment of the lungs on PET images. However, this acquisition reduces anatomical details and might miss small lung nodules in up to 34% of the cases [13, 14]. CT acquisition at mid-expiration is favoured [16]. The 4D CT approach was reported to enhance standardized uptake value (SUV) quantification by more than 50%, compared to mid-expiratory acquisition in malignant lesions [14, 15]. However, it involves higher radiation doses to the patient, and its implementation is not feasible in all centres.

Radiopharmaceutical-related artefacts can occur due to tracer injection, with eventual hot clots or extravasation, which could be interpreted as abnormalities [6]. Foci of increased uptake in the lungs not corresponding to a nodule or any other lung abnormality and axillary lymph nodes with increased uptake ipsilateral to the injection site with extravasation of the radiopharmaceutical are examples of radiopharmaceutical-related artefacts. Halo or photopenic artefacts formed in regions with a high tracer uptake, such as the kidneys, ureters, urinary catheters and bladder, might impede the correct interpretation of PET images. Halo artefacts can be caused by incorrect scatter correction (negative values near high activity regions) during image reconstruction (non-negativity constraint in statistical reconstruction) [11]. This artefact could result in misdiagnosis and often requires repeated scans, since it cannot be recovered using conventional algorithms [17, 18].

In the last few years, various deep-learning (DL)-based algorithms have been developed for quantitative PET image reconstruction [19–21]. Numerous ASC methodologies have been suggested, including scatter and attenuation estimation in the sinogram domain, indirect (attenuation map generation) and direct attenuation correction [6, 10, 22–29]. Furthermore, various DL algorithms have been proposed for artefact correction in single-modality PET, CT or MR imaging [6, 27, 28, 30, 31]. For example, we demonstrated in a previous study the potential of direct ASC framework for artefact correction in 2-deoxy-2-[^18^F]fluoro-D-glucose (^18^F-FDG) PET/CT [6, 10, 31]. In addition, DL-based metal artefact reduction in PET/CT in the image and projection domains showed that the DL algorithm outperformed conventional algorithms [32]. Another study successfully used DL for truncation and metallic artefact compensation in PET/MRI [33]. Ultimately, a more recent study investigated MR image artefact disentanglement using unpaired data and a DL algorithm [34].

DL algorithms commonly require a significant sample size for appropriate training [9, 28, 29, 35–40]. Moreover, building a robust DL model requires a large dataset involving a wide range of scanners, image acquisition and reconstruction protocols [9, 28, 29, 35–40]. However, data exchange across hospitals is restricted by ethical and regulatory considerations [9, 28, 29, 35–38]. In addition, all hospitals do not necessarily staffed by AI scientist, having access to computational resources for building centre-based models, and gathering large heterogeneous datasets remains challenging [9, 28, 29, 35–38]. Federated learning (FL) is a technique that allows DL models to be developed on distributed datasets spread across several hospitals or institutes [9, 29, 35–38, 41, 42]. FL aims to train a model that can draw knowledge from various decentralized datasets without moving the data to a single location [9, 29, 35–40]. This is especially helpful if the data are private or sensitive and cannot be shared with a central server or other hospitals [9, 29, 35–38, 43, 44].

There are several approaches to FL, including weighted federated averaging, which involves training a local model on each decentralized dataset and then averaging the model weights across all centres to create a global model [9, 29, 35–43]. In this scenario, the idea is to build one competitive global model with a centralized model [9, 29, 35–43]. Another approach referred to as federated transfer learning (FTL) involves a global model development through decentralized training and then fine-tuning the global model in each centre separately to create a centre-specific personalized model [9, 28, 29, 35–40]. FTL is useful in training DL models considering the high variability and heterogeneity of datasets across different centres, which requires large datasets and centre-specific models [9, 28, 29, 35–38].

As mentioned earlier, image artefacts remain a challenge in clinical molecular imaging, especially mismatch and halo artefacts in ^68^Ga PET imaging. Correcting these artefacts requires novel algorithmic developments since conventional algorithms have failed to address them adequately. In the current study, we employed differential privacy FTL to manage the data-sharing issue in the clinic for building DL models. In addition, we used FTL approaches to build centre-specific models with prior knowledge of different centres’ data that are specific to each centre. Furthermore, we employed this method for PET image artefact disentanglement on ^68^Ga-labelled compounds and evaluated its performance qualitatively and quantitatively. Finally, we compared the proposed method with centre-based and centralized algorithms.

## Materials and methods

### PET image acquisition and preprocessing

The institutional ethics committee of Geneva University Hospital approved this retrospective multi-institution study. This study enrolled a total of 1413 patients with ^68^Ga-prostate-specific membrane antigen (PSMA)/DOTA-TATE (TOC) PET/CT scans from 3 countries (Switzerland, Iran and Canada), scanned at 8 centres. An experienced nuclear medicine physician reviewed all images to select high-quality and artefact-free PET images for model development (421 clean data out of 1413). Table 1 provides details of the datasets from the different centres. CT-based ASC (CT-ASC) was used for PET image correction. Corrected and non-corrected PET images were converted to SUV units (units changed from Bq/ml to SUV) using injected dose, decay factor and patient’s weights [6, 9, 10, 28, 29]. All PET images were first cropped to the body contour and then zero-padded to the same bounding box (232 × 168 × *Z*, where *Z* is the slice number) to preserve both image resolution and body shape. All images were normalized between 0 and 1 using 90% of the histogram by dividing the value of non-ASC and CT-ASC by 2 and 5, respectively [6, 9, 10, 28, 29].

**Table 1:** Image acquisition and reconstruction settings in 8 different imaging centres

Centre	No	Clean	Train	Test	Scanner	Reconstruction	Tracers	Matrix size
Centre 1	70	16	12	4	Siemens Horizon	PSF + TOF + 3D-OSEM	^68^ Ga-PSMA	180 × 180
Centre 2	315	71	56	15	Siemens Biograph 6	3D-OSEM	^68^ Ga-PSMA	168 × 168
Centre 3	97	49	39	10	Siemens mCT	PSF + TOF + 3D-OSEM	^68^ Ga-PSMA	256 × 256
Centre 4	97	49	39	10	Siemens Vision	PSF + TOF + 3D-OSEM	^68^ Ga-PSMA	440 × 440
Centre 5	261	51	40	11	Siemens Biograph 6	PSF + 3D-OSEM	^68^ Ga-PSMA	168 × 168
Centre 6	295	57	45	12	Siemens mCT	3D-OSEM	^68^ Ga-PSMA	200 × 200
Centre 7	183	89	71	18	GE Discovery 690	3D-OSEM	^68^ Ga-DOTA-TATE	192 × 192
Centre 8	95	39	31	8	GE Discovery IQ	3D-OSEM	^68^ Ga-PSMA	192 × 192
Total	1413	421	333	88	-	-	^−^	-

### Deep neural network

In the current study, we implemented a modified version of the U2Net [9, 45]. U2Net architecture incorporates residual blocks and deep supervision [9]. In the U2Net architecture, each conventional U-Net block consists of U-Net network, comprising classical U-Net blocks, including convolution, batch normalization, ReLU activation function and up and down sampling with a symmetric encoder and decoder [9, 45]. The input of the deep neural networks in the different scenarios was non-ASC PET, with the target being CT-ASC to generate DL-ASC PET images as output automatically. The network was trained in 2D using the Adam optimizer with a learning rate of 0.001, L2-norm loss and a weight decay of 0.0001 [9, 45]. The network schema is shown in Supplemental Fig. 1. Artefact-free clean data sets were used for train, validation and test set.

**Fig. 1:**
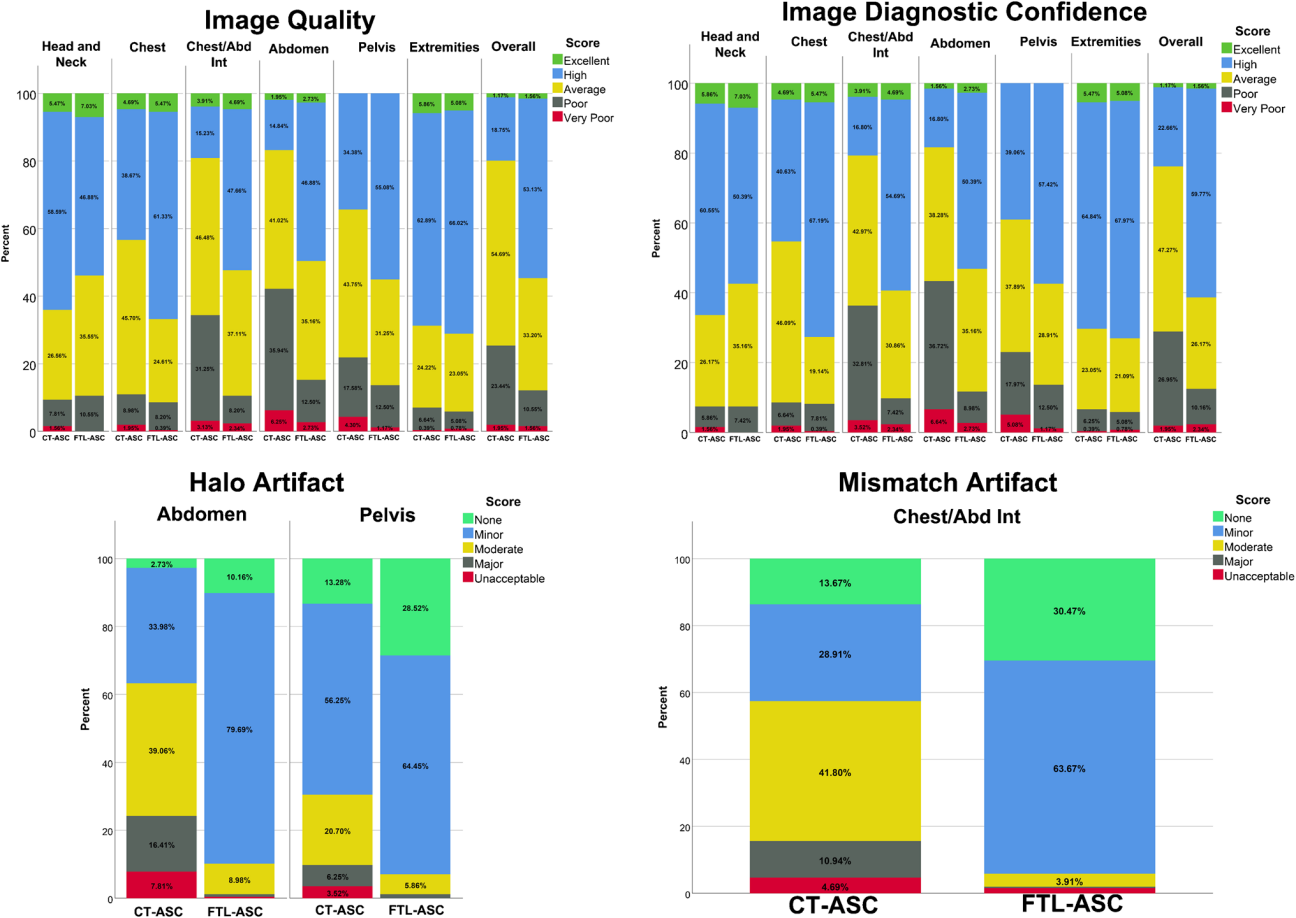
Image quality, image diagnostic confidence, halo and mismatch artefact comparison between CT-ASC and FTL-ASC in different regions of the body

### Different learning scenarios

Different scenarios were used to develop DL algorithms, including (i) centre-based (CeBa), (ii) centralized (CeZe) and (v) federated transfer learning (FTL).

#### CeBa

Each hospital independently developed the DL model using its own dataset [9, 28, 29, 35–42]. This training framework has trouble properly adjusting to unobserved new cases [9, 28, 29, 35–42]. Moreover, all hospitals do not necessarily have access to curated and large heterogeneous datasets, computational power and machine learning (ML) developers [9, 28, 29, 35–42].

#### CeZe

Different hospitals send data to a core hospital to create and develop a global ML model [9, 28, 29, 35–42]. This scenario jeopardizes the privacy of the data and hinders collaboration among different hospitals [9, 28, 29, 35–42].

#### FTL

The data are decentralized and not shared among hospitals in a FL framework, but the hospitals can work together and exchange DL model parameters to develop a global model [9, 28, 29, 35–42, 46, 47]. Each hospital keeps its own datasets, and the local datasets are used to develop the DL models independently [9, 28, 29, 35–38]. Each hospital sends model updates to a core hospital after training the local model. Then, the central server in the core hospital combines the model updates to create a global DL model. We implemented Gaussian differential privacy FL [9, 28, 29, 35–38, 48, 49] to build the global model using a decentralized data set and then fine-tuned the model for each hospital to develop personalized models for each hospital. More details were provided in the supplementary dataset. All models were implemented in Tensorflow and TensorFlow Federated (TFF) [50] using a server with multiple GPUs (RTX 2080 Ti) [9, 28, 29, 35–38]. The model trained on 80% of the clean data set (20% as the test set) at each hospital, and 10% of the training in each hospital (clean data) was used as a validation set to optimize the hyperparameters of the models.

### Evaluation strategies

Two different test sets were used for the evaluation of our proposed method. This includes an artefact-free clean test set (20% of each centre clean dataset) used for quantitative analysis and a second test set consisting of images presenting with artefacts (128 patients) for blind qualitative analysis.

### Quantitative analysis

Model performance was evaluated using image-derived PET metrics, including voxel-wise mean absolute error (MAE), mean squared error (MSE), structural similarity index (SSIM) and peak signal-to-noise ratio (PSNR) between ground truth CT-ASC and predicted DL-ASC PET images using artefact-free clean test set [10].

### Qualitative assessment of artefacted images

Two nuclear medicine experts blindly performed the qualitative analysis of 258 images from 128 cases. CT-ASC (128 images) and FLT-ASC (128 images) image assessments were performed blindly in terms of image artefacts (1: unacceptable, 2: mild, 3: moderate, 4: minor and 5: none), diagnostic confidence (1: very poor, 2: poor, 3: average, 4: high and 5: excellent) and image quality (1: very poor, 2: poor, 3: average, 4: high and 5: excellent). These analyses were performed separately for different body regions, including the head and neck (including the brain), chest, chest abdomen interval (diaphragm region), abdomen, pelvis and extremities, and whole images (all regions). Moreover, 30 images (15 from FLT-ASC and 15 from CT-ASC) were used to assess the intra-observer variability of each physician. Inter-observer variability was calculated across 256 images.

### Statistical analysis

Quantitative analyses were performed on the same clean test sets. Images with artefacts were used only for qualitative analysis. We used the Wilcoxon test to compare image-derived metrics between the different scenarios. The *p*-value was corrected using the false discovery rate correction method developed by Benjamin Hochberg [51] to provide an adjusted *p*-value (*q*-value). To assess consistency, we used the intraclass correlation coefficient (ICC) test based on a two-way mixed effects model for intra-/inter-observer variability assessment. ICC classified as poor reproducibility (ICC < 0.40), fair reproducibility (0.40 < ICC < 0.59), good reproducibility (0.60 < ICC < 0.74), or excellent reproducibility (0.75 < ICC < 1.00) [52]. The McNemar test was used for pairwise comparisons of image quality, artefacts and diagnostic confidence between CT-ASC and FLT-ASC images. The marginal homogeneity test compared these factors’ distributions between CT-ASC and FLT-ASC. We also used generalized linear models (GLMs) to adjust for reader and rate confounders, the centres and scanners and compare the distributions of image quality, artefacts and diagnostic confidence between CT-ASC and FLT-ASC in each region. Statistical analysis was performed using R software version 4.2.0, using a significance level of 0.05.

## Results

### Quantitative analysis of artefact-free images

#### Image-based analysis

The three approaches evaluated in this study for ^68^ Ga-PET imaging (CeBa, CeZe and FTL) resulted in MAE values of 0.42 ± 0.21 (*CI* 95%: 0.38 to 0.47), 0.32 ± 0.23 (*CI* 95%: 0.27 to 0.37) and 0.28 ± 0.15 (*CI* 95%: 0.25 to 0.31), respectively. Regarding SSIM, the FTL approach had a value of 0.80 ± 0.10 (*CI* 95%: 0.78 to 0.82). According to the Wilcoxon test, there were significant differences between the three approaches, with CeZe achieving 0.75 ± 0.15 (*CI* 95%: 0.72 to 0.79) and CeBa attaining 0.71 ± 0.15 (*CI* 95%: 0.68 to 0.74). The results showed that FTL-ASC outperformed CeBa and CeZe (*p*-value < 0.05). Supplemental Fig. 2 and Supplemental Table 1 compare the different metrics across different approaches in the test set. Supplemental Tables 2 and 3 provide more details for each centre for CeBa and FTL. We continued qualitative analysis of FTL-ASC PET images since FTL resulted in high quantitative accuracy.

**Fig. 2:**
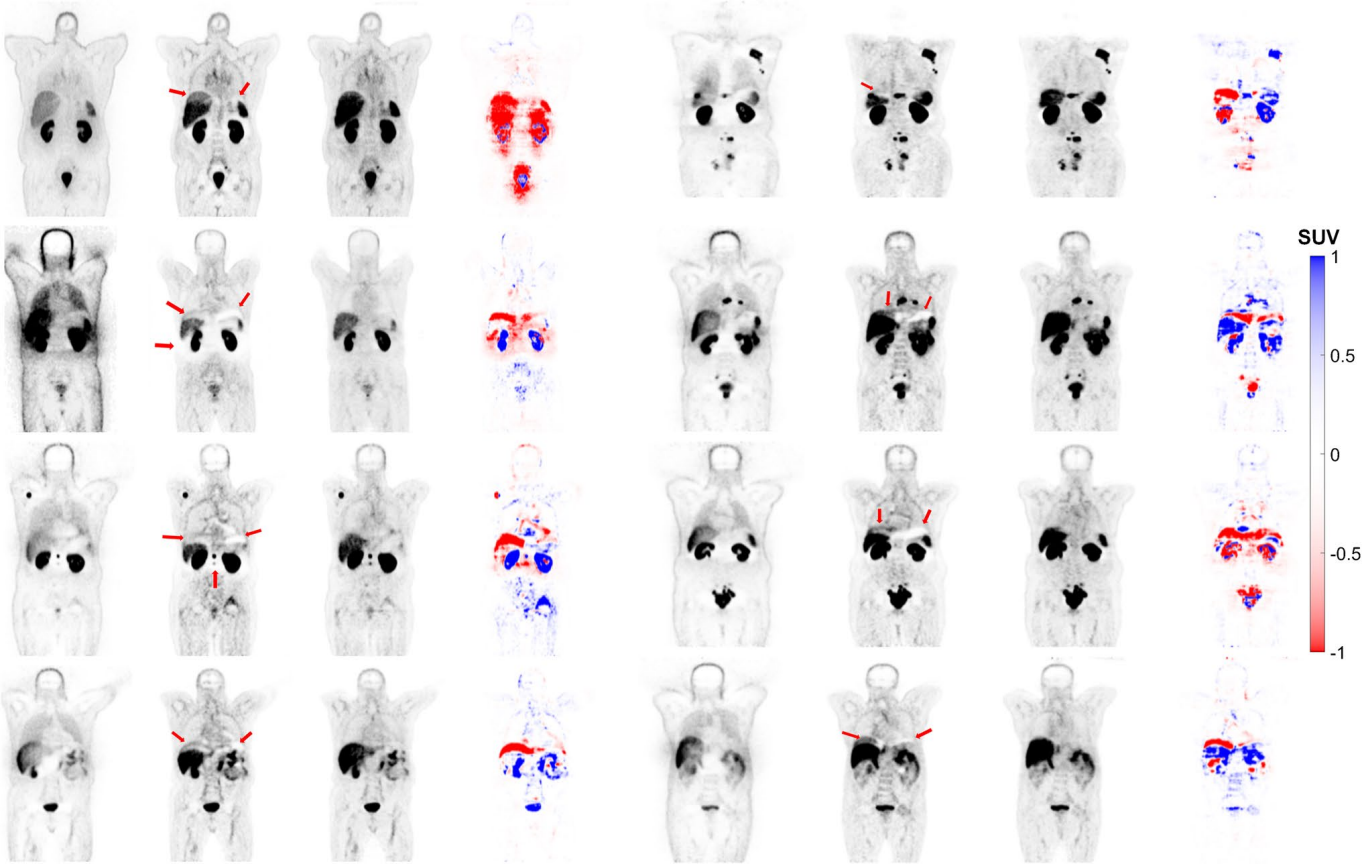
Coronal views of 8 representative clinical studies showing from left to right: non-ASC, CT-ASC, FTL-ASC and the difference images of CT-ASC and FTL-ASC. The images generated using the FTL approach successfully corrected the mismatch artefact in the diaphragm region (depicted by the arrows) and reduced photopenic artefacts in the lung, liver and spleen regions in the different cases

**Table 2:** Intra and inter-reader ICC for image quality, diagnostic confidence and image artefacts in all data for both readers

Intra and Inter-reader analysis			
Region	Image quality	Diagnostic confidence	Image artefact
Intra-Reader Analysis using 30 Images			
Head and neck	0.85	0.88	
Chest	0.71	0.79	
Chest abdomen interval	0.80	0.82	Motion: 0.86
Abdomen	0.73	0.76	Halo: 0.88
Pelvis	0.70	0.75	Halo: 0.82
Extremities	0.55	0.60	
All regions	0.72	0.76	
Inter-Reader Analysis using 256 Images			
Head and neck	0.66	0.66	
Chest	0.61	0.60	
Chest abdomen interval	0.72	0.74	Motion: 0.73
Abdomen	0.71	0.72	Halo: 0.82
Pelvis	0.60	0.61	Halo: 0.74
Extremities	0.41	0.47	
All regions	0.60	0.61

**Table 3:** Comparison of image quality, diagnostic confidence and image artefacts between CT-ASC and FLT-ASC. The reported *p*-value is based on a generalized linear model after adjustment of ICC for all raters (CT-ASC and FLT-ASC), readers and centres

Region	Image quality	Diagnostic confidence	Image artefact
Head and neck	0.566	0.711	-
Chest	0.062	**0.015**	**-**
Chest abdomen interval	** < 0.001**	** < 0.001**	Motion: < **0.005**
Abdomen	** < 0.002**	** < 0.001**	Halo: < **0.001**
Pelvis	0.211	0.211	Halo: < **0.05**
Extremities	0.801	0.801	-
All regions	**0.001**	**0.001**	**-**

Significant value mentioned in the text. *P*-value less than 0.05

### Qualitative assessment of artefacted images

#### Intra and inter-observer variability

The ICC values for image quality, artefacts and diagnostic confidence are shown in Table 2. Both inter- and intra-observer variability showed fair reproducibility in the extremities and good and excellent reproducibility in other regions of the body.

#### Overall image quality

Table 3 (generalized linear model tests) and Supplemental Tables 4–6 (McNemar and marginal homogeneity tests) compare the image quality, artefacts and diagnostic confidence between CT-ASC and FTL-ASC. In addition, the data are visualized in bar plots in Fig. 1. These results demonstrate that image quality improved significantly for the chest/abdomen and abdomen regions. In the pelvic region, image quality improved significantly from 34.38 to 55.08%, while very poor image quality significantly decreased from 4.3 to 1.17%. The overall image quality is significantly improved in all regions of the body (total image). Diagnostic image confidence was significantly improved in the chest, chest/abdomen and abdomen regions.

Moreover, high and very-poor diagnostic confidence in the pelvic region significantly increased and decreased, respectively, using FTL-ASC. FTL-ASC PET images significantly improved overall diagnostic confidence. The mismatch artefacts in the chest/abdomen region and halo artefacts in the abdomen and pelvis significantly decreased when using FTL-ASC.

#### Mismatch and halo artefacts

**Fig. 3:**
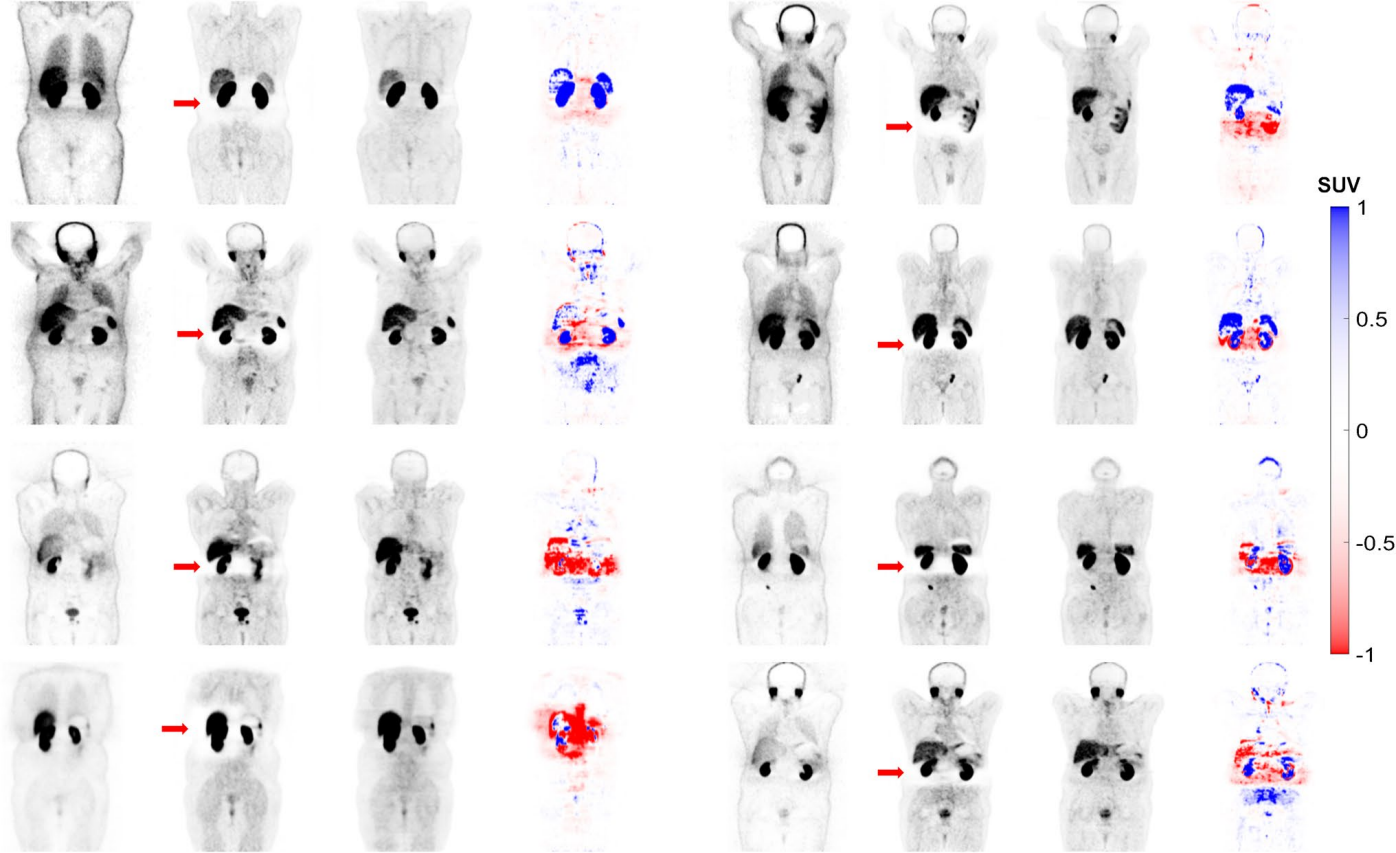
Coronal views of 8 representative clinical studies showing from left to right: non-ASC, CT-ASC, FTL-ASC and the difference images of CT-ASC and FTL-ASC. FTL-ASC effectively disentangles halo artefacts in the kidney area (depicted by the arrows) in the different cases

**Fig. 4:**
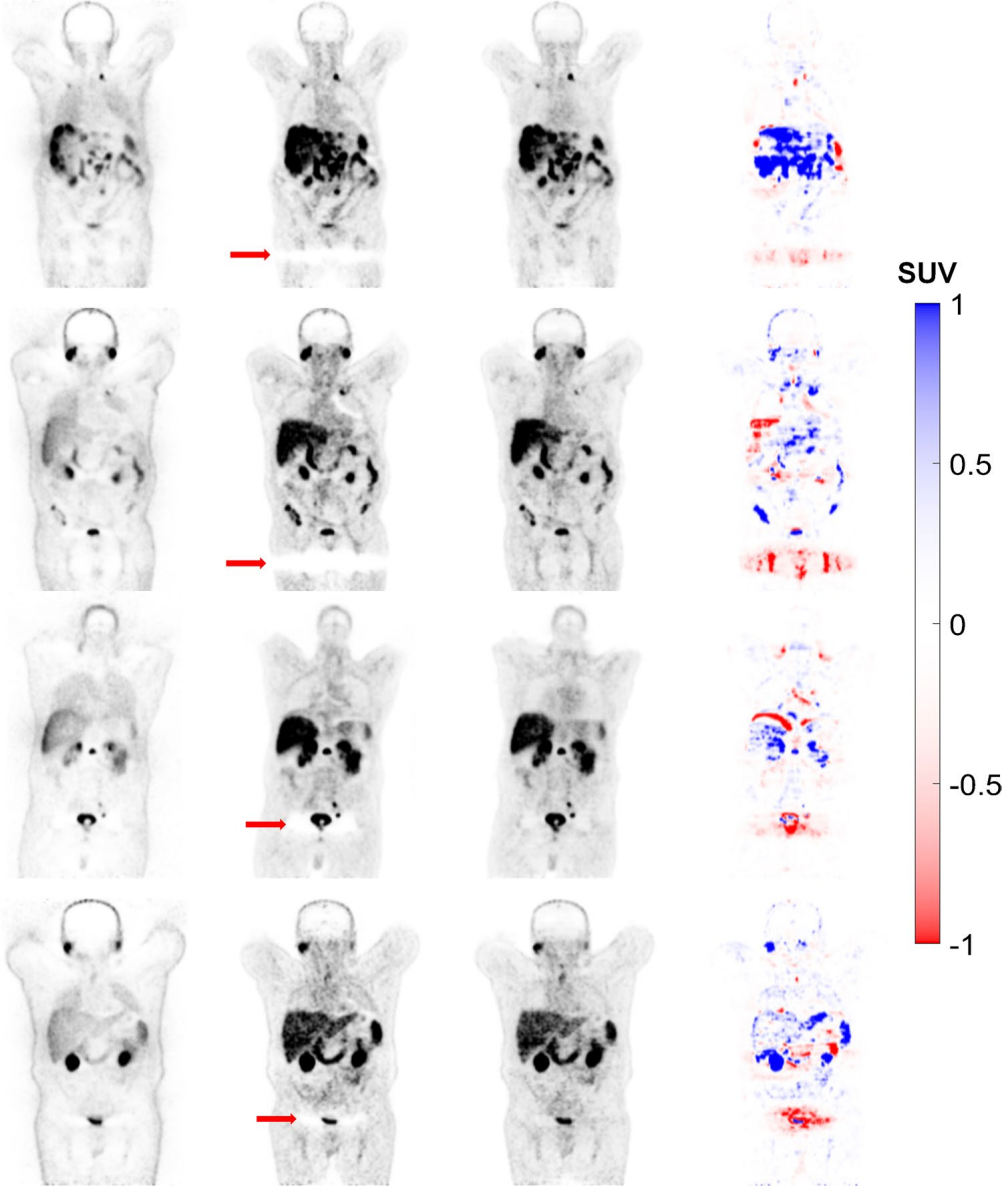
Coronal views of 4 representative clinical studies showing from left to right: non-ASC, CT-ASC, FTL-ASC and the difference images of CT-ASC and FTL-ASC. FTL-ASC effectively disentangles halo artefacts in the pelvic area (depicted by the arrows) in the different cases

The images generated using the FTL approach successfully corrected the mismatch artefact in the diaphragm region and reduced photopenic artefacts in the lung, liver and spleen region, as shown in Fig. 2. The results of our study demonstrate that FTL-ASC effectively disentangles halo artefacts in the abdominal and pelvic regions (Figs. 3 and 4).

Case study with repeated scans (upon request from the nuclear medicine physician immediately after the initial scan).

**Fig. 5:**
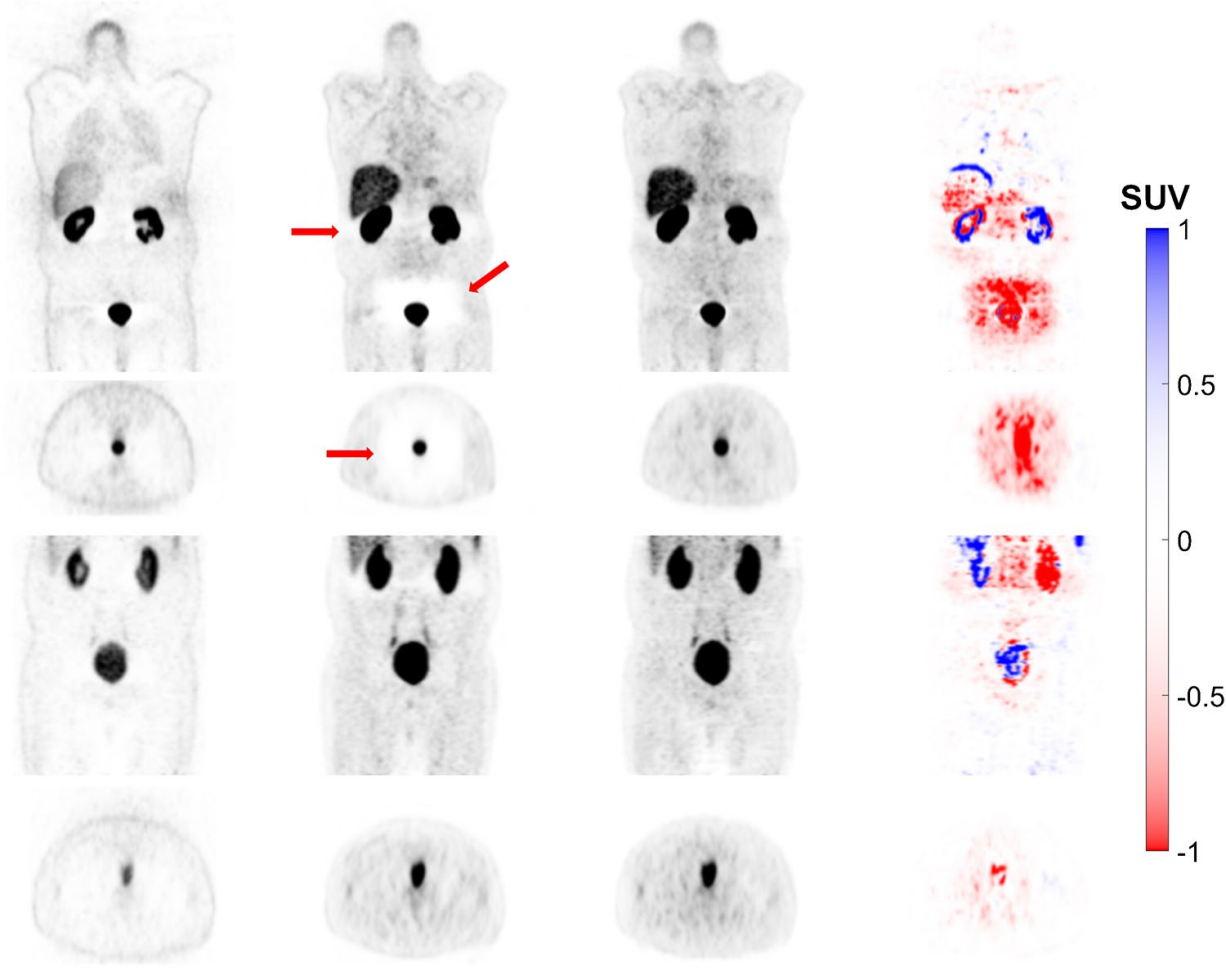
Coronal and axial views showing from left to right: non-ASC, CT-ASC, FTL-ASC and the difference images of CT-ASC and FTL-ASC. The top panel shows the initial scan, whereas the bottom panel depicts the repeated scan in the artefacted region (depicted by the arrows). In the repeated CT-ASC scan, this artefact was removed from the images. FTL-ASC produced high-quality artefact-free images in both scans

Figure 5 represents a patient with a halo artefact in the pelvic region and the repeated scan performed for this patient in the kidney and pelvic region. FTL-ASC shows high-quality artefact-free images in both scans. This artefact was removed in the repeated CT-ASC scan, and FTL-ASC PET images reported almost similar image quality, diagnostic confidence and pattern as the first scan.

**Fig. 6:**
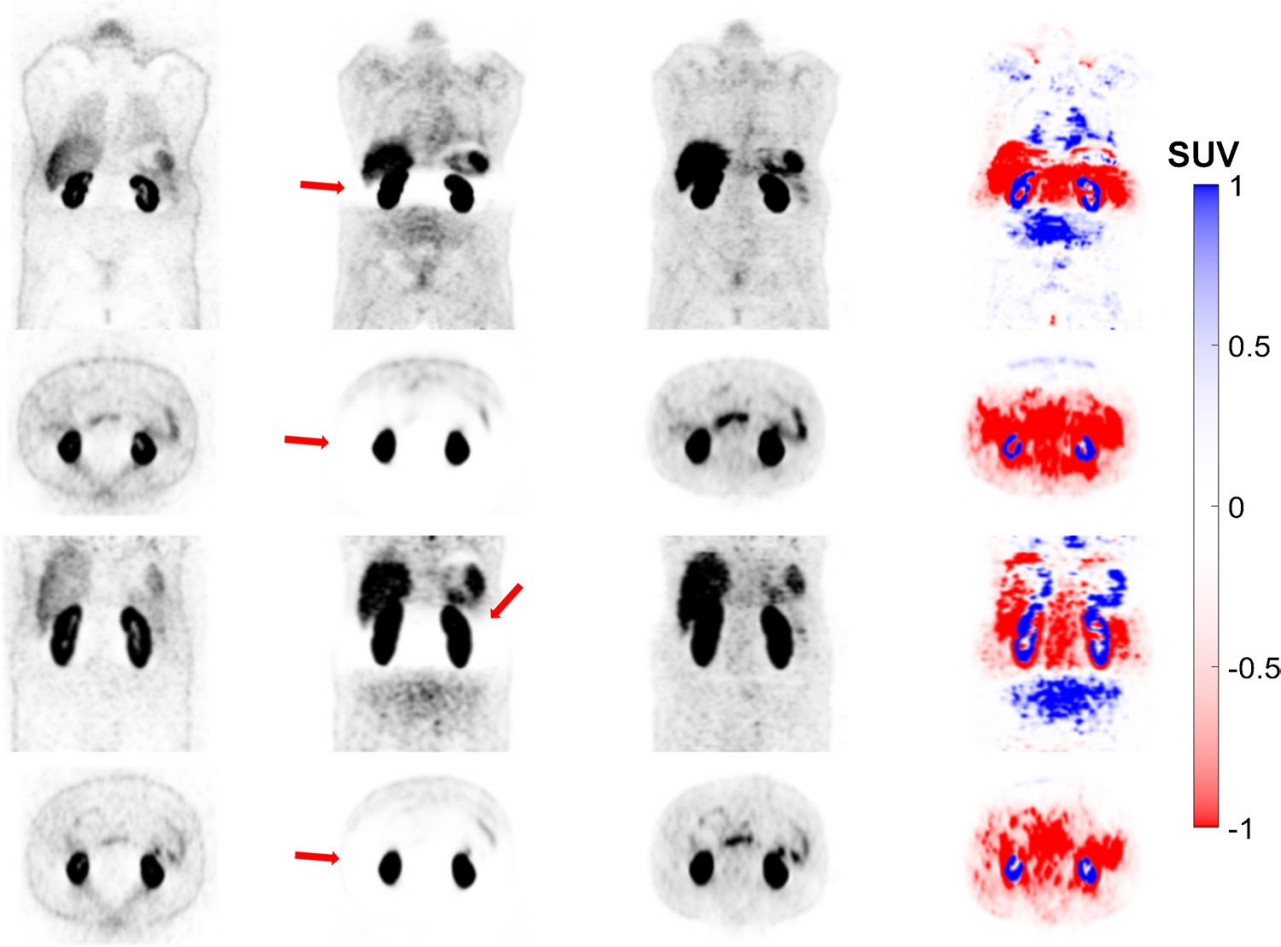
Coronal and axial views showing from left to right: non-ASC, CT-ASC, FTL-ASC and the difference images of CT-ASC and FTL-ASC. The top panel shows the initial scan, whereas the bottom panel depicts the repeated scan in the artefacted region (depicted by the arrows). The repeated scan could not remove this artefact, where the halo artefact remained visible in the same region. However, FTL-ASC successfully removed this artefact in both scans

Figure 6 shows a case with a halo artefact in the kidneys. The repeated scan was performed for this patient in this region owing to low image quality and diagnostic confidence. Unfortunately, the repeated scan could not remove this artefact. The halo artefact is still visible in the same region. However, the FTL-ASC model successfully removed this artefact in both scans.

**Fig. 7:**
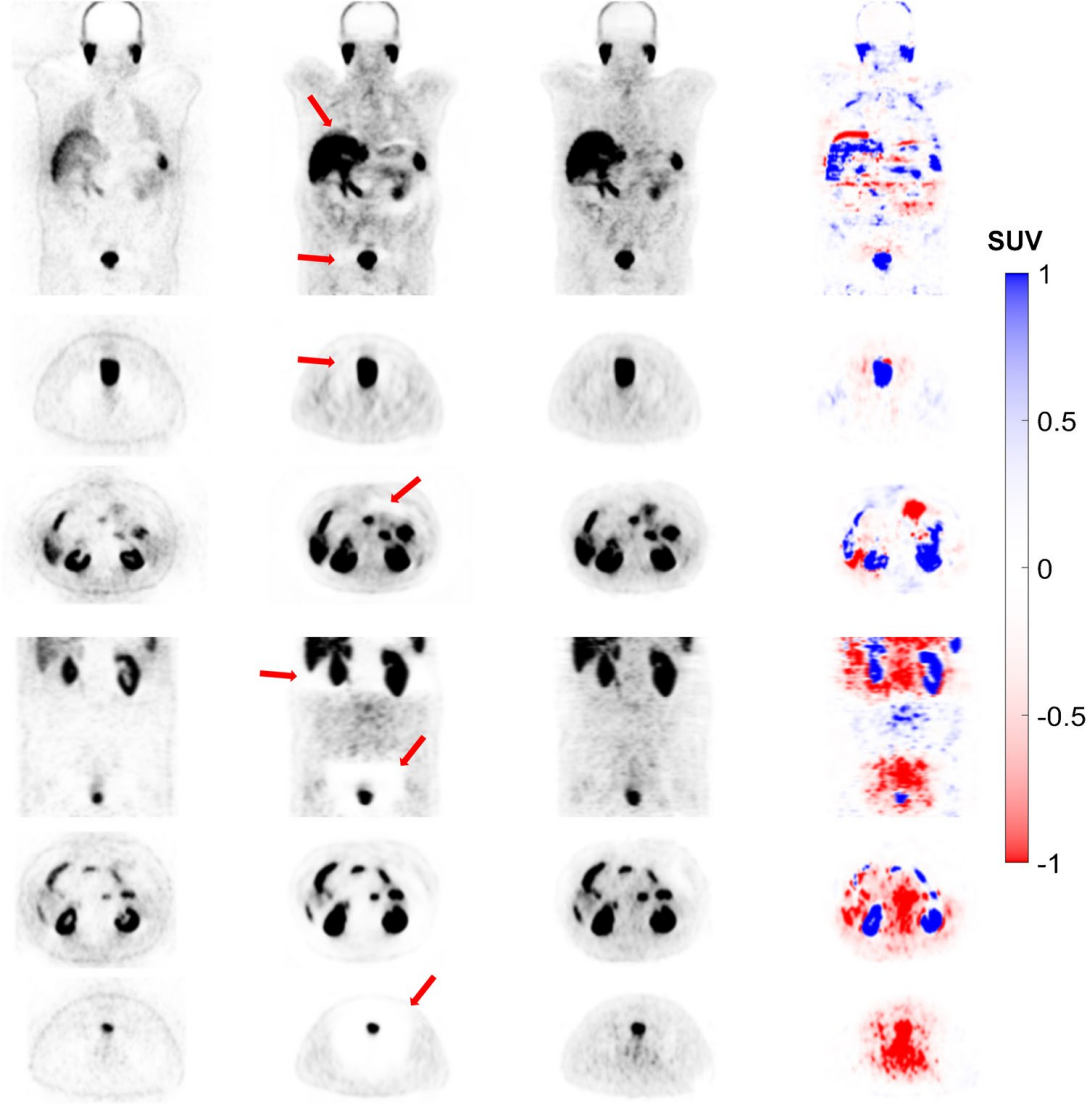
Coronal and axial views showing from left to right: non-ASC, CT-ASC, FTL-ASC and the difference images of CT-ASC and FTL-ASC. The top panel shows the initial scan, whereas the bottom panel depicts the repeated scan in the artefacted region (depicted by the arrows). The FTL-ASC PET image recovered high quality and high diagnostic confidence for both scans. The halo artefact was exaggerated and produced low-quality low diagnostic confidence images in the repeated scan

Figure 7 represents a patient with moderate halo artefacts in the abdomen and pelvic area, with the repeated scan performed for this patient. However, the repeated scan exaggerated the halo artefact resulting in low-quality and low diagnostic confidence images. The FTL-ASC PET image recovered high quality and high confidence for both scans.

## Discussion

A single universal model may not be effective due to differences in tracer injected activity, equipment, image acquisition and reconstruction strategies across different hospitals [9, 10, 28, 29, 35–42, 46, 47]. Therefore, it is necessary to create personalized models using large, heterogeneous datasets to overcome this issue [9, 28, 29, 35–38]. In the current study, we utilized differential privacy-preserving FTL to adopt a centralized model for each centre separately, resulting in improved accuracy for ASC in PET images and simultaneously addressing data-sharing privacy issues.

ASCs are the two major corrections based on CT images toward quantitative ^68^Ga PET imaging [6, 9, 10, 25, 28, 29]. However, mismatch and halo artefacts might appear on ^68^Ga PET images during this process, leading to potential changes in the diagnosis and prognosis of patients [11, 13, 14, 16–18]. In addition, these artefacts are difficult to detect and correct in real clinical scenarios [6]. The developed model does not require iterative image reconstruction incorporating ASC. In addition, we addressed the data-sharing issue using differential privacy FTL and showed that our model quantitatively outperformed centralized and centre-based models. We then used FTL-ASC for further qualitative analysis. Through quantitative analysis, we observe the impact of radiotracers and scanners on the performance of the models. We observed that FTL enhanced significantly the quantitative accuracy of the models in both situations, outperforming the CeBa approach. Different sources, such as scanner and radiotracer, significantly affect FTL’s effectiveness. Notably, FTL performs more efficiently when different scanners use the same radiotracer, rather than when diverse radiotracers are used on the same scanner. Qualitative analysis revealed the performance of our proposed model for effective mismatch and halo artefact detection and correction in the chest, abdomen and pelvic regions without knowledge of the ground truth in ^68^Ga PET images.

Different DL-based ASC methods have been proposed for PET, including indirect methods generating attenuation maps from MRI and non-ASC images or using maximum likelihood estimation of activity and attenuation (MLAA) and then using these attenuation maps for ASC during the reconstruction process [21, 27]. For example, Liu et al. [22] used a GAN network to generate pseudo-CT from PET-nonAC in brain PET images, whereas Dong et al. [23] used the same approach in whole-body imaging. In addition, Hwang et al. [24] improved the performance of MLAA by dealing with its main limitations (crosstalk artefacts, slow convergence speed and noisy attenuation maps) through DL for ^18^F-FDG brain PET and tested this methodology in whole-body PET imaging [25]. The direct DL-based ASC framework directly generates ASC PET images from non-ASC images [9, 10, 21]. Our group first implemented this approach in brain PET imaging [53]. Furthermore, our group [10] and Dong et al. [26] independently assessed the performance of direct ASC in whole-body ^18^F-FDG PET imaging.

A low injected tracer activity and the high positron range of ^68^Ga-labelled radiopharmaceuticals produce low-quality images, compared to ^18^F-labelled compounds, adding challenges to direct ASC [11, 13, 14, 16–18]. At first glance, direct ASC utilizing DL appears as an excessive use of artificial intelligence in PET. However, it not only generates quantitatively and qualitatively corrected PET images but also has some potential, which we emphasized in the current study [10]. The main potential of this approach is mismatch and halo artefact detection and correction of ^68^ Ga PET images without using anatomical images. In indirect techniques, reconstruction is mandatory to produce ASC PET images, potentially capable of addressing mismatch artefacts using a clean dataset [54, 55]. However, these approaches do not address halo artefacts, as this artefact appears during the reconstruction process and depends mostly on PET images (not CT images). Mismatch and halo artefacts were evaluated in the current study, where they were successfully detected and corrected in different regions of the body. We presented cases in which artefacts appeared in the original ^68^ Ga PET images, and FTL-ASC successfully removed these artefacts. Moreover, blind analysis performed by physicians showed significant improvement in image quality, confidence in diagnostic accuracy and removal of artefacts. In addition, we showed different scenarios in which repeated scans could be performed to remove these artefacts and observed that DL-based algorithms properly recovered important clinically relevant features. Meanwhile, there were a number of cases in which repeated scans could not remove and even exaggerated these artefacts. In this situation, our proposed DL-based algorithms could disentangle these artefacts without knowledge of the ground truth.

Most previous studies were performed on single-centre datasets, limiting the DL model generalizability [10, 22–26]. A multicentre study could potentially address this issue, where FL is definitely a good option. More recently, we developed a decentralized, federated DL model for ASC of ^18^F-FDG PET images using 300 clinical studies from 6 different centres [9]. We evaluated two different FL algorithms, parallel and sequential, without a privacy-preserving mechanism, such as the differential privacy implemented in the current study. We reported that both models outperformed the centre-based model and achieved performance levels comparable to the centralized model [9]. The differential privacy-preserving mechanism implemented in the current study can protect the model against various attacks, as FL by itself does not offer that protection [9]. One of the main advantages of FTL is the ability to create personalized DL models using data from various centres without compromising privacy. Furthermore, by enabling DL models to gain knowledge from a wider variety of data, FTL has the potential to enhance the performance of these models.

The use of FL poses additional challenges that need to be considered [39–42, 46, 47]. Decentralized datasets may be heterogeneous due to variations in data acquisition and reconstruction protocols across the different centres [9]. As a result, combining model updates and building a comprehensive global model might be challenging. In CeBa and CeZe training, the data set is small and highly heterogeneous, respectively [9, 36, 38]. In addition, a global FL model would achieve the accuracy of CeZe in the ideal scenario [9]. The current study used FTL algorithms to address the limitations of CeBa, CeZe and FL model development by building a global FL model in the first steps from the heterogeneous multicentric dataset and subsequently developing a centre-specific model using transfer learning [9]. FTL can be computationally demanding because it necessitates coordinating the training process across various centres, aggregating the model updates and providing a centre-specific model in each centre separately [36].

One of the main limitations of the current study is that model development took place on a single server equipped with multiple GPUs [9, 36, 38]. Future research should implement a more realistic computational model addressing the major challenges, such as the communication burden between different centres or the computation power of each centre [9, 36, 38]. Furthermore, various attacks should be designed to assess the performance of the differential privacy-preserving mechanism in protecting the privacy of data in different scenarios [9, 36, 38]. Additionally, since this method was developed based on direct ASC of ^68^Ga images, it is not suitable for other radiotracers, such as ^18^F-FDG, as it will fail to generate correct images due to domain and concept shifts [6, 9, 10, 29]. Further studies should be conducted to develop and evaluate the proposed artefact correction techniques for other tracers and other PET image artefacts [6, 9, 10, 29].

## Conclusion

We employed a differential privacy-preserving FTL framework for artefact detection and disentanglement in PET imaging of ^68^Ga-labelled compounds. The proposed approach benefits from using large datasets from multiple centres while preserving patient privacy. Simultaneously, it uses the transfer learning concept, providing site-specific models that outperform centralized and centre-based models. In addition, the qualitative analysis demonstrated that the proposed model correctly addresses two main challenging artefacts in ^68^Ga-PET imaging.

### Supplementary Information

Below is the link to the electronic supplementary material.

Supplementary file1 (PDF 348 KB)

## Data Availability

Trained models and code are available on GitHub.
