# Supplementary material for: Differential privacy preserved federated transfer learning for multi-institutional 68Ga-PET image artefact detection and disentanglement
Source: Eur J Nucl Med Mol Imaging. 2023 Sep 8;51(1):40–53. doi: 10.1007/s00259-023-06418-7 (PMC10684636; doi:10.1007/s00259-023-06418-7)
Supplement: Supplementary file 1 — Supplementary file1 (PDF 348 KB) [file 259_2023_6418_MOESM1_ESM.pdf]

Preliminaries of FL Building Blocks

Let us consider a network of K devices, each with a local dataset D_k for $k \in 1,2,\dots,K$. Each local dataset is composed of n_k examples, represented by tuples $\left(x_{k,i}, y_{k,i}\right)$ for $i\epsilon 1,2,\dots,n_k$ [1 - 6] . The goal of FTL is to train a global model f that can make predictions based on new examples given by (x, y), using the information from all the decentralized datasets D_k[1 - 6] . One way to perform FTL is through federated averaging [1-12]. This involves training a local model f_k on each decentralized dataset D_k, and then averaging the model weights across all the devices to create the global model:

$$f = \frac{1}{K}\sum_{k=1}^{K} f_k$$

Another approach is to use a federated learning server, which coordinates the training process across the decentralized datasets [1 - 6] . The server sends a global model f to each device, and the device uses its local data samples to compute model updates Δf_k [1 - 6] . The server then aggregates the updates to create a new global model:

$$f_{t+1} = \frac{1}{K}\sum_{k=1}^{K} \Delta f_k$$

One effective optimization technique for both local model training and model updating is stochastic gradient descent (SGD) [1-12]. The objective is to reduce the discrepancy between the predicted image or loss functions and the ground truth image [1 - 6] . After building the global model using the FL concept, the model is fine-tuned in each center separately to have knowledge from different centers and specified for each center separately (transfer learning) [1 - 6] .

Differential Privacy

By introducing noise to the data, differential privacy protects individuals' privacy within a dataset [3]. Differential privacy aims to ensure that the inclusion or exclusion of any individual from the dataset has no appreciable influence on the outcomes of statistical analysis [3, 13]. This is accomplished using a randomization mechanism, such as the Laplace or Gaussian mechanisms, to introduce noise into the data [3, 13-17]. The privacy budget ε, representing the maximum amount of privacy loss deemed acceptable, determines how much noise is added to the data [3]. The "sensitivity" of the function, also known as the difference in probability between any two outcomes, is a common way to define the privacy budget [13-17]. The maximum change in the function's output brought on by including or excluding a single subject in the dataset is referred to as the sensitivity [3, 13-17].

If algorithm M is randomized, it's considered (ϵ,δ)-differentially private when, for any two closely related datasets D1 and D2, and any specified event E in set R, the differences in the algorithm's output distributions for these datasets are within the bounds of $(e| \,|\epsilon, \delta)$[3, 13-17]. This means that, for any event E, the probability of the event occurring in the output distribution of the algorithm on the dataset $D_1$is no more than e^{ϵ} times the probability of the event occurring in the output distribution of the algorithm on the dataset D_2, plus δ [3]. If $\delta = 0$, and $\delta > 0$,the algorithm is termed pure differentially private (DP) and approximate DP, respectively [3].

The Gaussian noise mechanism is an effective technique for implementing DP [3, 13-17]. It adds zero-mean multivariate Gaussian noise with a standard deviation of $\sigma . \psi_f$, to the output of a function f with L_2-sensitivity ψ_f, which is defined as the maximum difference in the output of the function for any two

neighboring datasets [3]. The parameter σ is chosen based on ${\psi_f}^2$ and δ. Gaussian noise can be applied to local model parameters before server aggregation, to global parameters on the server before distribution, and during local training[3].

Supplemental Table 1. Summary statistics of quantitative parameters for different approaches.

	Approach	MAE	MSE	PSNR	SSIM
**Mean ± SD**	CeBa	0.42 ± 0.21	1.15 ± 2.03	24.76 ± 1.87	0.71 ± 0.15
	CeZe	0.32 ± 0.23	0.89 ± 2.41	25.85 ± 1.96	0.75 ± 0.15
	FTL	0.28 ± 0.15	0.63 ± 1.53	26.32 ± 1.85	0.80 ± 0.1
**CI95%**	CeBa	0.38 to 0.47	0.73 to 1.58	24.37 to 25.15	0.68 to 0.74
	CeZe	0.27 to 0.37	0.38 to 1.39	25.44 to 26.26	0.72 to 0.79
	FTL	0.25 to 0.31	0.31 to 0.95	25.94 to 26.71	0.78 to 0.82

Supplemental Table 2. Summary statistics of quantitative parameters for different centers trained for each center separately (CeBa) and tested on all test sets (centers 1-8). i.e., column Center 1 represents the results of testing on the whole test set when training is performed only using the Center 1 data set. All test sets represent the results of models, in which training and testing are performed at the same center (whole 20% of the clean dataset).

**Quantitative metric**	**Center 1**	**Center 2**	**Center 3**	**Center 4**	**Center 5**	**Center 6**	**Center 7**	**Center 8**	**All Test Set**
**MAE**	0.70 ± 0.27	0.52 ± 0.33	0.67 ± 0.29	0.56 ± 0.28	0.66 ± 0.19	0.55 ± 0.31	0.90 ± 0.41	0.73 ± 0.26	0.42 ± 0.21
**MSE**	2.45 ± 2.03	2.41 ± 2.69	2.94 ± 3.48	1.95 ± 2.22	2.83 ± 2.61	2.04 ± 2.32	3.03 ± 3.13	3.71 ± 2.93	1.15 ± 2.03
**PSNR**	22.90 ± 1.95	23.7 ± 2.80	22.6 ± 1.91	23.65 ± 2.01	22.7 ± 1.98	23.8 ± 2.44	22.3 ± 1.65	21.9 ± 1.82	24.76 ± 1.87
**SSIM**	0.61 ± 0.11	0.73 ± 0.17	0.66 ± 0.14	0.70 ± 0.15	0.56 ± 0.09	0.72 ± 0.16	0.53 ± 0.10	0.60 ± 0.12	0.71 ± 0.15
				**CI 95%**					
**MAE**	0.64 to 0.76	0.45 to 0.59	0.61 to 0.74	0.50 to 0.62	0.62 to 0.70	0.48 to 0.61	0.82 to 0.99	0.68 to 0.78	0.38 to 0.47
**MSE**	2.02 to 2.87	1.85 to 2.98	2.21 to 3.67	1.49 to 2.42	2.29 to 3.37	1.55 to 2.52	2.38 to 3.68	3.10 to 4.32	0.73 to 1.58
**PSNR**	22.50 to 23.30	23.10 to 24.30	22.20 to 23.00	23.20 to 24.10	22.20 to 23.10	23.30 to 24.30	22.00 to 22.70	21.50 to 22.30	24.37 to 25.15
**SSIM**	0.58 to 0.63	0.69 to 0.76	0.63 to 0.69	0.67 to 0.73	0.54 to 0.58	0.68 to 0.75	0.51 to 0.55	0.58 to 0.63	0.68 to 0.74

Supplemental Table 3. Summary statistics of quantitative parameters for the different centers using FTL and tested on all test sets (centers 1-8). i.e., column Center 1 represents the results of testing on the whole data set when training is performed only using the Center 1 data set. All test sets represent the results of models, in which training and testing are performed at the same center (whole 20% of the clean dataset).

Quantitative metric	Center 1	Center 2	Center 3	Center 4	Center 5	Center 6	Center 7	Center 8	All Test Set
**MAE**	0.41 ± 0.20	0.33 ± 0.18	0.37 ± 0.22	0.33 ± 0.15	0.33 ± 0.15	0.37 ± 0.19	0.62 ± 0.36	0.33 ± 0.17	0.28 ± 0.15
**MSE**	1.05 ± 1.66	0.8 ± 1.70	1.16 ± 2.74	0.70 ± 1.59	0.74 ± 1.46	0.89 ± 1.52	2.07 ± 3.03	0.84 ± 1.83	0.63 ± 1.53
**PSNR**	25.00 ± 1.96	25.77 ± 1.91	25.00 ± 1.86	25.87 ± 1.64	25.74 ± 1.85	25.30 ± 1.76	23.49 ± 1.97	25.63 ± 2.02	26.32 ± 1.85
**SSIM**	0.73 ± 0.09	0.78 ± 0.13	0.71 ± 0.16	0.78 ± 0.12	0.77 ± 0.12	0.75 ± 0.15	0.72 ± 0.08	0.78 ± 0.12	0.80 ± 0.10
					**CI 95%**				
**MAE**	0.37 to 0.45	0.29 to 0.36	0.33 to 0.42	0.29 to 0.36	0.29 to 0.36	0.33 to 0.4	0.55 to 0.70	0.29 to 0.36	0.25 to 0.31
**MSE**	0.70 to 1.40	0.44 to 1.15	0.58 to 1.73	0.37 to 1.03	0.43 to 1.04	0.57 to 1.21	1.44 to 2.71	0.46 to 1.22	0.31 to 0.95
**PSNR**	24.59 to 25.41	25.37 to 26.17	24.61 to 25.39	25.53 to 26.22	25.35 to 26.13	24.93 to 25.67	23.08 to 23.90	25.21 to 26.05	25.94 to 26.71
**SSIM**	0.71 to 0.75	0.75 to 0.80	0.68 to 0.75	0.75 to 0.80	0.75 to 0.80	0.72 to 0.78	0.70 to 0.73	0.75 to 0.81	0.78 to 0.82

Supplemental Table 4. Comparison of image quality between CT-ASC and FLT-ASC The P-value is based on the McNemar test; P-value$ is based on the marginal homogeneity test.

**Region**	**Image Quality**	**P-value**	**P-value$**
**Head and Neck**	Excellent	0.640	0.793
	High	0.071	
	Average	0.107	
	Poor	0.421	
	Very poor	0.990	
**Chest**	Excellent	0.604	0.151
	High	<**0.001**	
	Average	<**0.001**	
	Poor	0.690	
	Very poor	0.990	
**Chest Abdomen Interval**	Excellent	0.990	**<0.001**
	High	<**0.001**	
	Average	0.184	
	Poor	<**0.001**	
	Very poor	0.990	
**Abdomen**	Excellent	0.990	**0.001**
	High	<**0.004**	
	Average	0.421	
	Poor	<**0.001**	
	Very poor	0.150	
**Pelvis**	Excellent	0.999	0.324
	High	<**0.001**	
	Average	**0.026**	
	Poor	0.254	
	Very poor	**0.045**	
**Extremities**	Excellent	0.992	0.979
	High	0.583	
	Average	0.780	
	Poor	0.990	
	Very poor	0.990	
**All regions**	Excellent	0.990	**0.002**
	High	<**0.001**	
	Average	<**0.001**	
	Poor	<**0.001**	
	Very poor	0.990	

Supplemental Table 5. Comparison of diagnostic confidence between CT-ASC and FLT-ASC. The P-value is based on the McNemar test; P-value$^{\$}$ is based on the marginal homogeneity test.

**Region**	**Diagnostic confidence**	**P-value**	**P-value$^{\$}$**
**Head and Neck**	Excellent	0.640	0.901
	High	0.121	
	Average	0.107	
	Poor	0.640	
	Very poor	0.990	
**Chest**	Excellent	0.641	**0.021**
	High	**<0.001**	
	Average	**<0.001**	
	Poor	0.640	
	Very poor	0.990	
**Chest Abdomen Interval**	Excellent	0.990	**<0.001**
	High	**<0.001**	
	Average	**0.049**	
	Poor	**<0.001**	
	Very poor	0.990	
**Abdomen**	Excellent	0.472	**<0.001**
	High	**<0.001**	
	Average	0.788	
	Poor	**<0.001**	
	Very poor	0.150	
**Pelvis**	Excellent	0.999	0.333
	High	**0.003**	
	Average	0.158	
	Poor	0.254	
	Very poor	**0.002**	
**Extremities**	Excellent	0.990	0.993
	High	0.578	
	Average	0.754	
	Poor	0.990	
	Very poor	0.990	
**All regions**	Excellent	0.990	**0.002**
	High	**<0.001**	
	Average	**<0.001**	
	Poor	**<0.001**	
	Very poor	0.990	

Supplemental Table 6. Comparison of artifact between CT-ASC and FLT-ASC. The P-value is based on the McNemar test; P-value$ is based on the marginal homogeneity test.

Region	Artifact	P-value	P-value$
**Chest Abdomen Interval-Motion**	None	**<0.001**	**0.009**
	Minor	**<0.001**	
	Moderate	**<0.001**	
	Major	**<0.001**	
	Unacceptable	0.990	
**Abdomen-Hallo**	None	0.995	**<0.001**
	Minor	**<0.001**	
	Moderate	**<0.001**	
	Major	**0.003**	
	Unacceptable	**0.031**	
**Pelvis-Hallo**	None	**<0.001**	**<0.001**
	Minor	**0.002**	
	Moderate	**<0.001**	
	Major	0.150	
	Unacceptable	**0.048**	

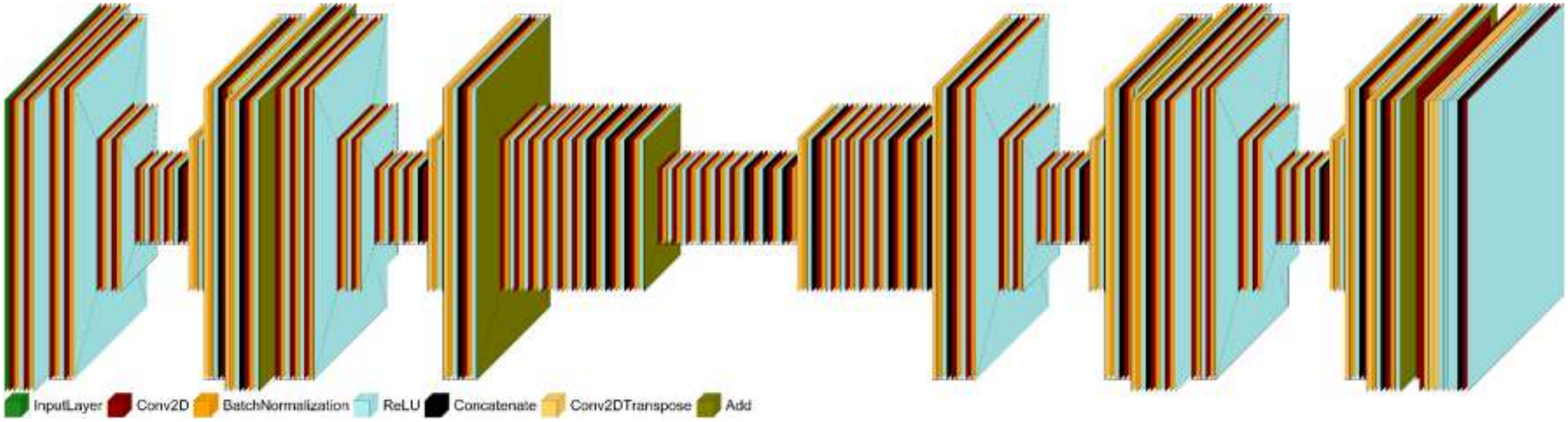

Supplemental Figure 1. Neural network architecture implemented in the current study.

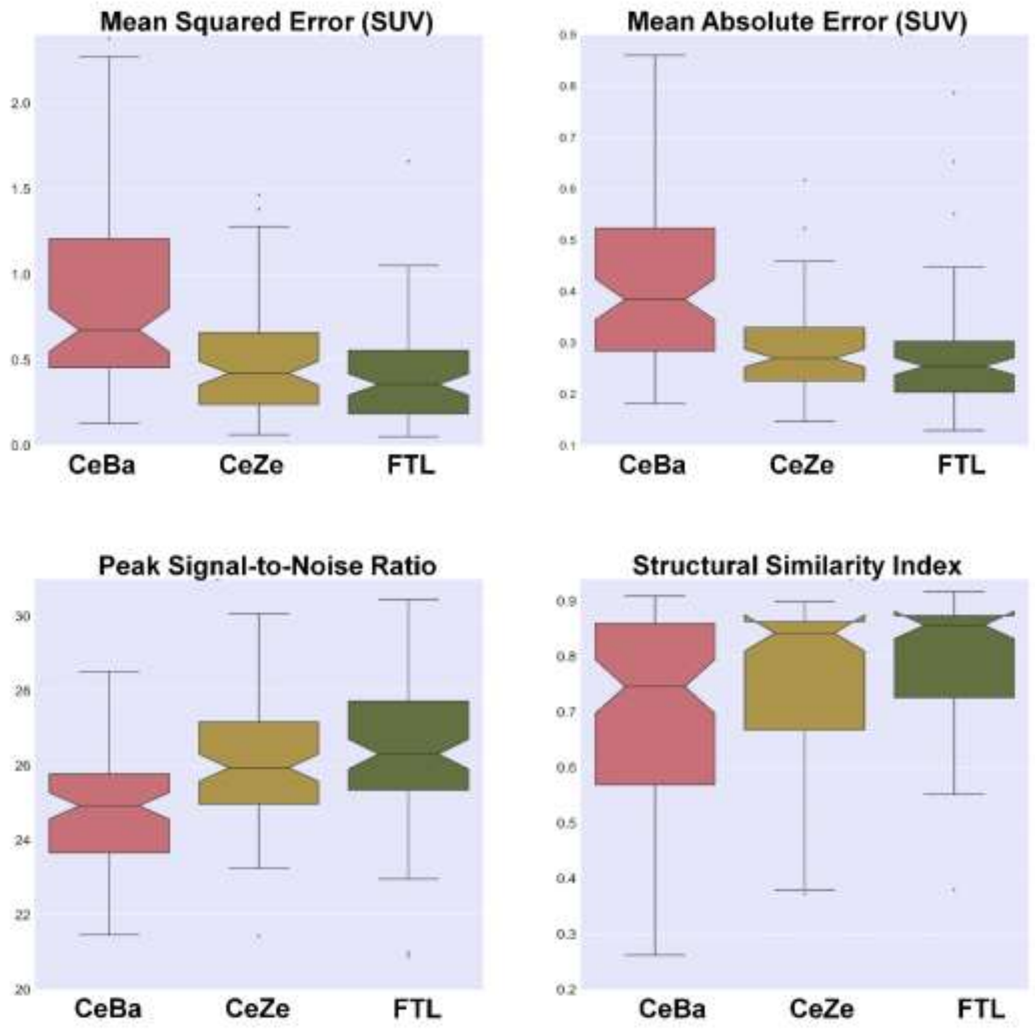

Supplemental Figure 2. Comparison between various scenarios using different quantitative metrics.

References

1. Shiri I, Vafaei Sadr A, Akhavan A, Salimi Y, Sanaat A, Amini M, et al. Decentralized collaborative multi-institutional PET attenuation and scatter correction using federated deep learning. Eur J Nucl Med Mol Imaging. 2023;50:1034-50. doi:10.1007/s00259-022-06053-8.
2. Shiri I, Vafaei Sadr A, Amini M, Salimi Y, Sanaat A, Akhavanallaf A, et al. Decentralized Distributed Multi-institutional PET Image Segmentation Using a Federated Deep Learning Framework. Clin Nucl Med. 2022;47:606-17. doi:10.1097/rlu.0000000000004194.
3. Shiri I, Razeghi B, Vafaei Sadr A, Amini M, Salimi Y, Ferdowsi S, et al. Multi-institutional PET/CT image segmentation using federated deep transformer learning. Comput Methods Programs Biomed. 2023;240:107706. doi:10.1016/j.cmpb.2023.107706.
4. Shiri I, Sadr AV, Sanaat A, Ferdowsi S, Arabi H, Zaidi H. Federated learning-based deep learning model for PET attenuation and scatter correction: a multi-center study. 2021 IEEE Nuclear Science Symposium and Medical Imaging Conference (NSS/MIC): IEEE; 2021. p. 1-3.
5. Shiri I, Amini M, Salimi Y, Sanaat A, Saberi A, Razeghi B, et al. Multi-institutional PET/CT image segmentation using a decentralized federated deep transformer learning algorithm. Soc Nuclear Med; 2022.
6. Shiri I, Showkatian E, Mohammadi R, Razeghi B, Bagheri S, Hajianfar G, et al. Collaborative Multi-Institutional Prostate Lesion Segmentation from MR images Using Deep Federated Learning Framework.
7. Rieke N, Hancox J, Li W, Milletarì F, Roth HR, Albarqouni S, et al. The future of digital health with federated learning. NPJ Digit Med. 2020;3:119. doi:10.1038/s41746-020-00323-1.
8. Kaissis GA, Makowski MR, Rückert D, Braren RF. Secure, privacy-preserving and federated machine learning in medical imaging. Nature Machine Intelligence. 2020;2:305-11.
9. Rahman KJ, Ahmed F, Akhter N, Hasan M, Amin R, Aziz KE, et al. Challenges, applications and design aspects of federated learning: A survey. IEEE Access. 2021;9:124682-700.
10. Shyu C-R, Putra KT, Chen H-C, Tsai Y-Y, Hossain KT, Jiang W, et al. A systematic review of federated learning in the healthcare area: From the perspective of data properties and applications. Applied Sciences. 2021;11:11191.
11. Rahman SA, Tout H, Talhi C, Mourad A. Internet of things intrusion detection: Centralized, on-device, or federated learning? IEEE Network. 2020;34:310-7.
12. AbdulRahman S, Tout H, Ould-Slimane H, Mourad A, Talhi C, Guizani M. A survey on federated learning: The journey from centralized to distributed on-site learning and beyond. IEEE Internet of Things Journal. 2020;8:5476-97.
13. Wei K, Li J, Ding M, Ma C, Yang HH, Farokhi F, et al. Federated learning with differential privacy: Algorithms and performance analysis. IEEE Transactions on Information Forensics and Security. 2020;15:3454-69.
14. Liu Z, Guo J, Yang W, Fan J, Lam K-Y, Zhao J. Privacy-preserving aggregation in federated learning: A survey. IEEE Transactions on Big Data. 2022.
15. Andrew G, Thakkar O, McMahan B, Ramaswamy S. Differentially private learning with adaptive clipping. Advances in Neural Information Processing Systems. 2021;34:17455-66.
16. Chen W-N, Choo CAC, Kairouz P, Suresh AT. The fundamental price of secure aggregation in differentially private federated learning. International Conference on Machine Learning: PMLR; 2022. p. 3056-89.
17. Li Q, Wen Z, Wu Z, Hu S, Wang N, Li Y, et al. A survey on federated learning systems: vision, hype and reality for data privacy and protection. IEEE Transactions on Knowledge and Data Engineering. 2021.
